# Characteristics of Wellens' Syndrome in the Current PCI Era: A Single-Center Retrospective Study

**DOI:** 10.1155/2023/8865553

**Published:** 2023-03-24

**Authors:** Li Zhou, Xuhe Gong, Hui Chen, Tianhui Dong, He-he Cui, Hongwei Li

**Affiliations:** Department of Cardiology, Cardiovascular Center, Beijing Friendship Hospital, Capital Medical University, Beijing 100050, China

## Abstract

**Objectives:**

The goal of this retrospective study was to reveal the prevalence, angiographic characteristics, clinical presentation, and long-term outcomes of non-ST-segment elevation myocardial infarction (NSTEMI) patients with Wellens' syndrome.

**Background:**

Procedural results for percutaneous coronary intervention (PCI) in acute coronary syndrome (ACS) have improved in recent years. However, there is still a paucity of available clinical trial data for Wellens' syndrome even though it is a well-known high-risk ACS.

**Methods:**

Among a total of 3528 patients with ACS who underwent angioplasty from 2017 to 2019 at the Cardiovascular Center of Beijing Friendship Hospital, 476 NSTEMI patients with culprit left anterior descending (LAD) vessels were enrolled in this study. According to electrocardiographic criteria of Wellens' syndrome, the patients were divided into a Wellens group (*n* = 138) and a non-Wellens group (*n* = 338). The primary endpoint was cardiac death; the secondary endpoints were main adverse cardiovascular and cerebrovascular events (MACCEs), a composite of all-cause death, cardiac death, heart failure, target lesion revascularization, recurrent myocardial infarction, and stroke. All of the medical and follow-up data were obtained from our institutional database.

**Results:**

The incidence of Wellens' syndrome in all ACS patients was 5.7% (200 of 3528). Among the 200 patients with Wellens' syndrome, 138 had NSTEMI, for a proportion of 69%. There was a significant decrease in the percentage of preexisting coronary heart disease (CHD), prior myocardial infarction, and previous PCI (*P* < 0.05) in the Wellens group compared with the non-Wellens group. On coronary angiography, single-vessel lesions were more common in the Wellens group (11.6% vs. 5.3%, *P*=0.016), and almost all (97.1%) of these patients received drug-eluting stents. Notably, the Wellens group had a higher proportion of early PCI than the non-Wellens group (71% vs. 61.2%, *P*=0.044). At 24 months, there was no statistically significant difference in cardiac death (*P*=0.111) between the two groups, but the MACCEs were comparable (Wellens: 5.1% vs. non-Wellens: 13.3%, *P*=0.009). Age ≥65 years was the largest independent risk factor for adverse prognosis.

**Conclusions:**

With early recognition and aggressive intervention, Wellens' syndrome is no longer a risk factor for adverse prognosis in patients with NSTEMI in the current PCI era.

## 1. Introduction

Acute coronary syndrome (ACS) is one of the leading causes of acute chest pain, requiring emergency care and eventual hospitalization [1–3]. An accurate assessment of the clinical setting is needed to ensure that the patient is correctly managed. This includes a good anamnesis, physical examination, and electrocardiographic (ECG) and cardiac biomarker evaluation. However, within the spectrum of ACS, subtle presentations cannot be overlooked. Wellens' syndrome is one such example, which in ACS patients is a catastrophic event often accompanied by extensive anterior myocardial infarction and high mortality rates. Wellens' syndrome, first described by Wellens and his group in 1982 [4], is the characteristic ECG pattern of T waves in the precordial leads that are associated with critical stenosis in the left anterior descending (LAD) artery. Early recognition and appropriate intervention for this syndrome carry significant diagnostic and prognostic consequences [5]. According to the Fourth Universal Definition of myocardial infarction [6], absence of ST-elevation in the precordial leads and the symmetrical and often deep (>2 mm) T wave inversions in the anterior precordial leads are an early sign that may precede the elevation of the ST-segment [7]. Thus, this syndrome was thought to be an acute ST-segment elevation myocardial infarction (STEMI) equivalent [8].

In previous studies [4, 9], Wellens' syndrome mainly presented with unstable angina pectoris (UAP). Actually, it should be noted that with the implementation of cardiac troponin (cTn) and high-sensitivity cardiac troponin (hs-cTn) assays, the diagnosis has been revolutionized [10]. Among the patients with Wellens' syndrome, the incidence of unstable angina is decreasing, and the diagnosis of non-ST-segment elevation myocardial infarction (NSTEMI) is increasing. Prompt identification of patients with Wellens' syndrome is critical. These individuals constitute a special cohort with their own clinical characteristics, which may affect the outcomes in this population. However, only limited data have been published regarding this syndrome, in which the authors reported mostly sporadic cases and clinical experience. Sparse studies of large sample sizes are available on patients with Wellens' syndrome. Updated information on the incidence, risk factors, angiographic findings, and prognosis of this subset of patients should be taken into consideration when taking care of these patients. The goal of this retrospective control study was therefore to investigate the risk factor profile, angiographic and clinical characteristics, and long-term outcomes of patients with Wellens' syndrome vs. other NSTEMI patients with culprit LAD vessels who were admitted to Beijing Friendship Hospital in China between 2017 and 2019.

## 2. Methods

### 2.1. Study Design and Participants

**Figure 1:**
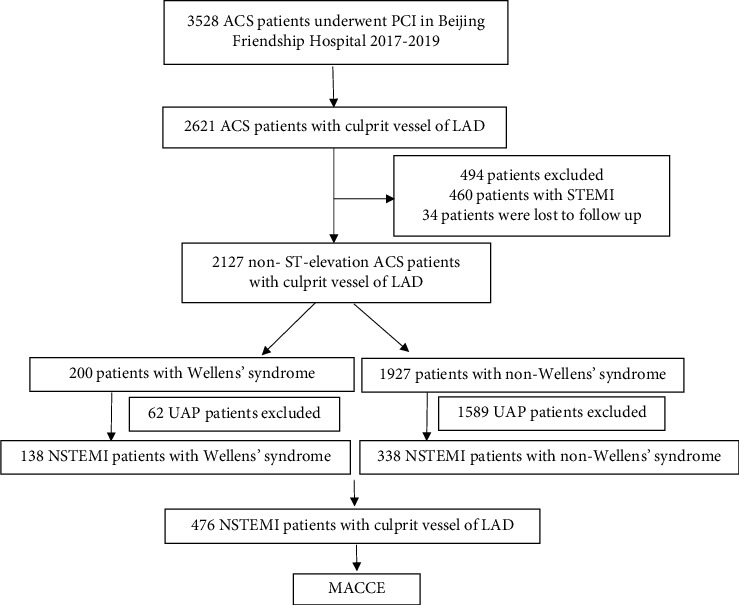
Flowchart of patient enrollment.

From January 2017 to December 2019, coronary angioplasty was performed in 3528 consecutive ACS patients at Cardiovascular Center, Beijing Friendship Hospital, and a total of 2621 patients with culprit LAD vessels were enrolled in this study. The medical retrospective data were collected and recorded in CBD-BANK (Cardiovascular Center Beijing Friendship Hospital Database Bank) supported by the DHC Software system (Dong Hua Software Co., Ltd). Both 460 STEMI patients and 1651 UAP patients were excluded. In addition, 34 patients were lost to follow-up. Finally, a total of 476 NSTEMI patients were included in the final analysis, all of whom were shown angiographically to have significant LAD stenosis. Among these patients, 138 patients met the ECG criteria of Wellens' syndrome and served as the Wellens group. The remaining 338 patients were assigned to the non-Wellens group. A flowchart of patient enrollment is shown in Figure 1.

Standard 12-lead ECGs were routinely obtained from all patients at the time of presentation to the emergency department and were reviewed by two independent cardiologists. Additional ECGs were measured during and after new episodes of chest pain. According to ECG pattern and angiography, the criteria for Wellens' syndrome are as follows [11, 12]: (a) symmetric and deeply inverted T waves, in leads V2 and V3, occasionally in leads V1, V4, V5, and V6, or biphasic T waves in leads V2 and V3, (b) isoelectric or minimally elevated (1 mm) ST segment, (c) no pathological precordial Q waves, (d) no loss of precordial R waves, (e) history of angina, and (f) angiographically significant LAD stenosis.

### 2.2. Data Collection and Follow-Up

Baseline data, including demographic information, initial clinical presentation (waist circumference and blood pressure at admission), cardiovascular risk factors, and past medical history, such as hypertension, diabetes, dyslipidaemia, chronic kidney disease (CKD), peripheral arterial disease (PAD), heart failure (HF), smoking, preexisting coronary heart disease (CHD), and previous PCI, were collected from CBD-BANK and then analysed. Laboratory examination results including haemoglobin A1c (HbA1c), creatinine, and lipid spectra were collected from CBD-BANK and then analysed. Echocardiography (ECHO) was performed (Philips IE33) for routine parameters, such as left ventricular ejection fraction (LVEF) and end-diastolic dimension (EDD), at baseline. The characteristics of coronary artery lesions and stent implantation information were evaluated by angiographic and PCI procedures and are presented in the medical documents.

After discharge, all patients were followed up for at least 24 months. Regular follow-up was conducted by clinic visits or phone interviews every 1–3 months. All information was recorded in the data bank.

### 2.3. Primary and Secondary Endpoints

The primary endpoint for this analysis was cardiac death, defined as previously reported, including death from myocardial infarction, heart failure, or arrhythmia, as well as unexplained sudden death [13]. A composite of major adverse cardiovascular and cerebrovascular events (MACCEs), including all-cause death, cardiac death, heart failure, target lesion revascularization, recurrent myocardial infarction, and stroke during follow-up, was defined as the secondary study endpoint. Clinically driven target lesion revascularization was defined as any repeat percutaneous intervention of the target lesion or bypass surgery of the target vessel that was performed for restenosis or other complication of the target lesion [14]. Recurrent myocardial infarction was defined by the Fourth Universal Definition [6]. All MACCEs were confirmed by two separate cardiologists simultaneously. In addition, readmissions due to cardiogenic causes such as hypertension, angina pectoris, myocardial infarction, arrhythmia, and cardiac insufficiency were included in the clinical outcomes.

All procedures performed in studies involving human participants were in accordance with the Institutional Ethics Committee of the Beijing Friendship Hospital affiliated with Capital Medical University (2021-P2-096-01) and with the 1964 Declaration of Helsinki and its later amendments or comparable ethical standards. This retrospective study was considered minimal risk by the Institutional Ethics Committee; therefore, formal consent was not needed.

### 2.4. Statistical Analysis

Continuous variables are expressed as mean ± standard deviation or median with interquartile range, and one-way analysis of variance was used to compare differences between continuous variables. Categorical variables are expressed as percentages and were analysed using Pearson's *χ*^2^ test or Fisher's exact test of variance. The cumulative incidence was estimated by the Kaplan‒Meier method, and differences between groups were assessed by the log-rank test. A two-sided *P* value <0.05 was considered statistically significant.

Cox regression was used to estimate the relative risk among groups of patients. All factors showing significance in the univariate analysis (*P* < 0.05) or an indicator clinically considered important for the outcome was then examined by multivariate analysis. All analyses were performed by using SPSS (version 25.0, Chicago, IL, USA); Kaplan–Meier survival curves were generated with GraphPad Prism software (version 5; GraphPad, Inc., San Diego, CA) [15].

## 3. Results

### 3.1. Baseline Characteristics

A total of 200 patients presented with Wellens' syndrome. The prevalence of this syndrome was 5.7% (200 of 3528) in the study population. In addition to 138 NSTEMI patients, there were 62 cases diagnosed as UAP. Out of the 476 NSTEMI patients enrolled, as mentioned above, 29% (*n* = 138) had Wellens' syndrome, and 71% (*n* = 338) had culprit LAD vessels only but did not satisfy the ECG criteria. They were divided into a Wellens group and a non-Wellens group. The clinical characteristics of the study cohort are described in Table 1. The two groups had similar demographics of age, sex distribution, smoking history, hypertension, and hyperlipidaemia. There were also no significant differences in the medical history of HF, CKD, PAD, and stroke. When compared to the non-Wellens group, the proportions of history of CHD (21.7% vs. 34.9%, *P* = 0.005), prior myocardial infarction (10.1% vs. 18.6%, *P* = 0.022), diabetes mellitus (26.8% vs. 42%, *P* = 0.002), and previous PCI (9.4% vs. 18.9%, *P* = 0.011) were much lower in Wellens patients. Among the other risk factors, lower levels of HbA1c (6.43 ± 1.52 vs. 6.84 ± 1.62, *P* = 0.011) and BMI (24.99 ± 3.12 vs. 25.66 ± 3.37 kg/m^2^, *P* = 0.045) were detected in Wellens patients. Nevertheless, there were no significant differences in EDD and LVEF.

Table 2 shows the differences in angiographic characteristics and treatment data between Wellens and non-Wellens patients. Among the two groups, single-vessel disease, i.e., involving only the LAD, was much higher in the Wellens group than in the non-Wellens group (11.6% vs. 5.3%, *P*=0.016). Left main (LM) disease prevalence tended to be lower in the Wellens group (10.9% vs. 18%, *P*=0.052), but there were no significant differences. In terms of angioplasty strategies, Wellens patients had more drug-eluting stents implanted (97.1% vs. 89.6%, *P*=0.007) but fewer plain balloon dilatations (2.9% vs. 9.2%, *P*=0.017). There were no remarkable differences in length (<30 mm, 31.9% vs. 34.6%, *P*=0.38) or number of stents (1.37 ± 0.64 vs. 1.28 ± 0.7, *P*=0.176). Notably, the proportion of early PCI (first medical contact to balloon ≤ 24 h) was significantly increased in the Wellens group (71% vs. 61.23%, *P*=0.044).

### 3.2. Clinical Outcomes

**Figure 2:**
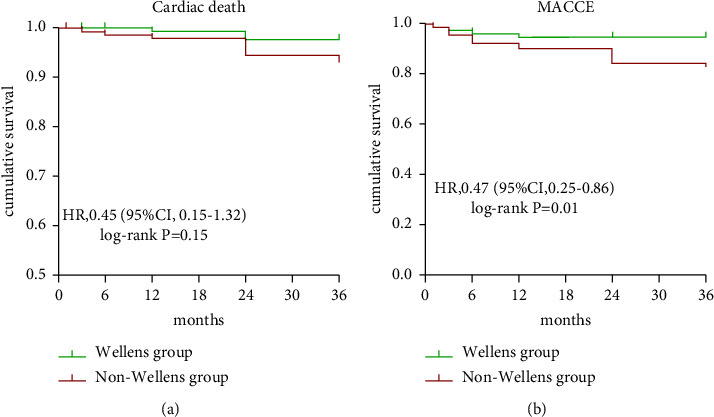
Kaplan–Meier analysis of MACCE (a) and cardiac death (b) for overall patients stratified by Wellens' syndrome (green line) and non-Wellens' syndrome (red line).

Up to the 24-month median follow-up, the clinical outcomes in the Wellens and non-Wellens groups are shown in Table 3. There were no significant differences in the primary endpoint (cardiac death, Wellens: 1.4% vs. non-Wellens: 4.4%, *P*=0.111). In addition, differences in all-cause death, HF, target vessel revascularization, recurrent myocardial infarction, and stroke were also not detected between the two groups. However, the Wellens group had significantly lower rates of composite MACCEs (5.1% vs. 13.3%, *P*=0.009) and cardiac readmission (8.7% vs. 23.1%, *P* < 0.001) than the non-Wellens group. Kaplan‒Meier curves also illustrated the incidences of primary (cardiac death, *P* log-rank = 0.15) and secondary endpoints (MACCE, *P* log-rank = 0.01) of the two groups in detail (Figure 2). For the secondary endpoint, there was a significant reduction in MACCEs in the Wellens group compared to the non-Wellens group.

### 3.3. Predictors of Survival

**Figure 3:**
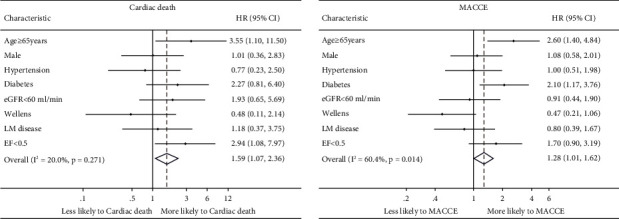
Factors independently associated with cardiac death and MACCE in overall patients in multivariable Cox regression analysis.

Considering all patients, a multivariable Cox regression analysis (Table 4) was used to identify clinical and angiographic independent predictors of cardiac death and MACCE. For cardiac death, the final multivariable mode included age ≥65 years and EF <0.5. In terms of MACCEs, the final multivariable model included age ≥65 years and diabetes mellitus. Overall, Wellens' syndrome was not associated with an increased risk of cardiac death (*P*=0.334) or MACCE (*P*=0.07). In Figure 3, age ≥65 years was the largest predictive factor of cardiac death (HR: 3.55, 95% CI: 1.10–11.50) and poor MACCEs (HR: 2.60, 95% CI: 1.40–4.84).

## 4. Discussion

Wellens' syndrome, also known as LAD coronary T-wave syndrome or “widow maker,” is observed in a subset of patients who often present with chest pain and are found to have specific precordial T-wave changes on ECG. Its prevalence is estimated to be approximately 10–15% of acute coronary syndrome (ACS) cases. The natural course of this pattern is potentially dangerous, with a high incidence of recurrent symptoms. It can rapidly progress to an anterior wall myocardial infarction if left untreated, causing catastrophic outcomes due to lesions in the proximal LAD artery. In Wellens' initial study group, of 145 patients admitted for UAP, 9% had the typical pattern upon presentation, with a further 9% developing T-wave changes within 24 h [9]. If they were treated medically without revascularization, approximately seventy-five percent of these patients developed anterior myocardial infarction within 1 week, despite the temporary relief of symptoms. Generally, Wellens' T-wave changes mainly occur during a pain-free interval when other evidence of ischaemia or infarction may be absent [16]. So, the Wellens sign was often formerly overlooked at hospital visits. However, as awareness of this syndrome has increased, early recognition and aggressive intervention have become the primary choice for physicians.

For Wellens' syndrome, cardiac biomarkers can be falsely reassuring, as they are typically normal or only minimally elevated. Previous data suggest that only 12% of patients with this syndrome have elevated cardiac biomarker levels, and the levels are always less than twice the upper limit of normal [9]. However, among 2127 NSTE-ACS patients with culprit LAD vessels in our study, 200 patients (9.4%) presented with Wellens' syndrome, including 138 patients with NSTEMI and 62 patients with UAP. Unlike prior studies, our research demonstrated that Wellens' syndrome more often manifests as NSTEMI than UAP (69% vs. 31%). This increased incidence rate of NSTEMI could be due to the adoption of the Fourth Universal Definition of Myocardial Infarction, which was based on elevated cardiac troponin. On the other hand, the former type of patient is usually pain-free at the time of presentation, and his or her cardiac biomarker concentration returns to the normal reference range. However, most patients will immediately seek medical attention for chest pain, and in such cases, a significant rise in cardiac biomarkers is frequently detected.

The mechanism of Wellens' syndrome remains unclear. In Wellens' sign, the ST segment and the first half of the T wave are essentially normal. T-wave inversion is generally seen hours or days after myocardial ischaemic pain subsides. During pain, T waves are usually upright with ST elevation or ST depression [17]. The T-wave change is not a sign of acute coronary occlusion but rather a sign of coronary artery reperfusion that is very likely to reocclude [18, 19]. There is also a view that the changes in the ECG account for the reperfusion of the ischaemic myocardium due to the alleviation of spasm of the proximal LAD artery [20, 21]. Other views, such as the lack of adaptive response, remain to be discussed.

Multiple cardiovascular risk factors are common in patients with Wellens' syndrome, and the majority of these patients are revealed to have at least one typical cardiovascular risk factor. Our study found that the proportion of DM was much lower in Wellens group, but there was no significant difference in other traditional risk factors between the two groups. However, in terms of past medical history, Wellens patients were much less likely than non-Wellens patients to have a history of CHD, MI, and previous PCI at admission, which reveals that Wellens' syndrome tends to occur in patients with new-onset cardiovascular disease.

The angiographic and procedural characteristics of patients with Wellens' syndrome may differ from the presentation in other NSTEMI patients. Compared with the non-Wellens group, the Wellens group had fewer LM patients and more single-vessel disease, with more than twice as many single-vessel patients. More Wellens than non-Wellens patients were treated with drug-eluting stents for revascularization, and fewer Wellens patients were treated with balloons only. This discrepancy may, to a large degree, be explained by a higher percentage of proximal and middle LAD lesions in Wellens patients. Balloon angioplasty, including both plain-old balloon angioplasty and drug-coated balloon angioplasty, is not appropriate for these critical sites [22, 23].

The prognosis of ACS patients depends on the earliest recognition of the symptoms along with subtle clues on the ECG, which would lead the physician to stratify the patients into low-risk, medium-risk, or high-risk categories and thus to act accordingly. Stabilization of the plaque by medical management in low-risk patients or revascularization by coronary angioplasty mostly in medium-risk/high-risk patients is the primary requirement. As a known high-risk group, Wellens patients had a greater proportion of early PCI than did non-Wellens patients in our study. These data indicate that cardiologists are more inclined to apply aggressive interventions for patients with Wellens' syndrome in clinical practice.

As previously discussed, patients with Wellens' syndrome have a substantial risk for future cardiovascular events, including extensive anterior wall infarction, malignant arrhythmias, cardiogenic shock, and sudden death. Somewhat unexpectedly, there was no statistically significant difference in cardiac death between the two groups during the follow-up period. Moreover, the 2-year incidence of MACCEs was much lower in the Wellens group than in the non-Wellens group (5.1% vs. 13.3%, *P*=0.009). These results support the view that patients with Wellens' syndrome may have a favorable prognosis in the long run. A possible explanation is that this low incidence was achieved by our appropriate management, which aggressively treated Wellens patients with urgent angiography and intervention. As mentioned earlier, the proportion of early PCI was higher in the Wellens group in our study. This highlights the importance of timely identification of Wellens' syndrome and aggressive invasive strategies in this group of patients. Most patients, when identified early and taken for cardiac catheterization, do well after appropriate intervention. Regarding the strength of the improvement in medical procedures, a small number of Wellens patients (8.7%) were readmitted within 2 years after PCI, which was much fewer than those from the non-Wellens group (23.1%, *P* < 0.001). Another possible explanation for the lower cardiac readmission incidence is that more patients with Wellens' syndrome have single-vessel coronary artery disease, which allows them to achieve complete revascularization during the first hospitalization. Recent randomized trials and meta-analyses have demonstrated the prognostic benefit of complete revascularization in NSTEMI patients by reducing unplanned repeat revascularization, all-cause mortality, cardiac mortality, and recurrent infarction [24–26]. It is worth mentioning that age ≥65 years was the largest independent risk factor for cardiac death and MACCEs in this study, while Wellens' syndrome was a protective factor against adverse prognosis.

Early PCI is currently the preferred treatment for patients with high-risk NSTE-ACS. In our real application, once a patient was diagnosed with Wellens' syndrome and enzyme elevation, he or she was admitted through the fast-track process. After an initial evaluation, the patient would undergo cardiac catheterization as soon as possible. We presumed that an early invasive strategy could attenuate the risk of MACCE and avoid long-term adverse outcomes in patients with Wellens' syndrome.

## 5. Limitations

Various limitations of the current analysis should be acknowledged. First, it was an observational study with potential bias and unmeasured confounding factors of nonrandomized analyses. Second, we only included patients who underwent coronary angioplasty, and thus the generalizability of our findings is limited. Moreover, the choice of therapeutic strategy reflected the convention and tendency of our single center, which may have affected the objectivity of the conclusions. Further prospective multicenter studies are needed to validate our findings.

## 6. Conclusions

First, the present study revealed that the incidence of Wellens' syndrome can reach up to 5.7% as assessed by coronary arteriography in clinical practice and 9.4% in NSTE-ACS patients with culprit LAD vessels. Second, Wellens' syndrome more often occurs in people with new-onset CHD, with NSTEMI having become the predominant clinical manifestation in these patients. Third, single-vessel coronary artery disease is more common in patients with Wellens' syndrome than in other NSTEMI patients. Although still regarded as an ominous omen, Wellens' syndrome is no longer a risk factor for adverse prognosis in the current PCI era.

## Figures and Tables

**Table 1:** Comparison of the baseline characteristics among patients.

Variable	Wellens (*n* = 138)	Non-Wellens (*n* = 338)	*P*
Age (years)	63 ± 10.5	64.6 ± 10.7	0.137
Male	108 (78.3)	257 (76)	0.602
Days	5.75 ± 9	6 ± 9	0.085
BMI (kg/m^2^)	24.99 ± 3.12	25.66 ± 3.37	**0.045**
WC (cm)	90.02 ± 9.95	91.66 ± 9.79	0.106
Smoke	88 (63.8)	211 (62.4)	0.783
HT	90 (65.2)	238 (70.4)	0.266
DM	37 (26.8)	142 (42)	**0.002**
Hyperlipidaemia	70 (50.7)	177 (52.4)	0.745
CHD	30 (21.7)	118 (34.9)	**0.005**
Prior MI	14 (10.1)	63 (18.6)	**0.022**
HF	1 (0.7)	6 (1.8)	0.388
CKD	6 (4.3)	23 (6.8)	0.309
PAD	8 (5.8)	27 (8)	0.406
Stroke	32 (16.7)	62 (18.3)	0.665
History of PCI	13 (9.4)	64 (18.9)	**0.011**
EDD (cm)	5.21 ± 0.57	5.28 ± 0.6	0.255
LVEF	0.61 ± 0.09	0.6 ± 0.1	0.063
LDL-C (mmol/L)	2.6 ± 0.71	2.51 ± 0.86	0.29
eGFR (ml/min)	87.69 ± 23.94	83.56 ± 26.93	0.118
HbA1c (%)	6.43 ± 1.52	6.84 ± 1.62	**0.011**

Data are presented as absolute numbers and percentages (for categorical variables) or mean value ± SD (for continuous variables) unless otherwise specified. BMI, body mass index; WC, waist circumference; HT, hypertension; DM, diabetes mellitus; CHD, coronary heart disease; MI, myocardial infarction; HF, heart failure; CKD, chronic kidney disease; PAD, peripheral arterial disease; PCI, percutaneous coronary intervention; EDD, left ventricular end-diastolic dimension; LVEF, left ventricular ejection fraction; LDL-C, low-density lipoprotein cholesterol; eGFR, estimated glomerular filtration rate (calculated via Modification of Diet in Renal Disease equation); HbA1c, glycated haemoglobin. Bold values indicate that the difference is statistically significant (*P* < 0.05).

**Table 2:** Angiographic characteristics and treatment data of patients.

Variable	Wellens (*n* = 138)	Non-Wellens (*n* = 338)	*P*
Single-vessel disease	16 (11.6)	18 (5.3)	**0.016**
LM disease	15 (10.9)	61 (18)	0.052
Plain balloon angioplasty	4 (2.9)	31 (9.2)	**0.017**
Drug-eluting stent	134 (97.1)	303 (89.6)	**0.007**
Stent length < 30 mm	44 (31.9)	121 (34.6)	0.38
Number of stents used	1.37 ± 0.64	1.28 ± 0.7	0.176
Early PCI (FMC to B ≤ 24 h)	98 (71)	207 (61.2)	**0.044**

Values are expressed as *n* (%), mean ± SD, or median with interquartile range. FMC to B, first medical contact to balloon. Bold values indicate that the difference is statistically significant (*P* < 0.05).

**Table 3:** Comparison of clinical outcome between Wellens and non-Wellens groups.

Event, *n* (%)	Wellens (*n* = 138)		Non-Wellens (*n* = 338)	*P*
MACCEs	7 (5.1)		45 (13.3)	**0.009**
All-cause death	3 (2.2)		21 (6.2)	0.068
Cardiac death	2 (1.4)		15 (4.4)	0.111
Heart failure	1 (0.7)		10 (3)	0.141
Target vessel revascularization	0		3 (0.9)	0.267
Recurrent myocardial infarction	3 (2.2)		20 (5.9)	0.084
Stroke	2 (1.4)		7 (2.1)	0.651
Cardiac readmission	12 (8.7)		78 (23.1)	**<0.001**

MACCE: major adverse cardiac and cerebrovascular events, a composite of cardiac death, heart failure, target vessel revascularization, recurrent myocardial infarction, and stroke.

**Table 4:** Multivariate Cox regression analysis in the overall patients.

	Predictor variable	HR (95% CI)	*P*
Cardiac death	Age ≥ 65 years	3.55 (1.10–11.5)	**0.035**
	Male	1.01 (0.36–2.83)	0.990
	HT	0.77 (0.23–2.50)	0.660
	DM	2.27 (0.81–6.4)	0.121
	eGFR <60	1.93 (0.65–5.69)	0.236
	EF <0.5	2.94 (1.08–7.97)	**0.034**
	Wellens' syndrome	0.48 (0.11–2.14)	0.334
	LM disease	1.18 (0.37–3.75)	0.776

MACCE	Age ≥ 65 years	2.6 (1.40–4.84)	**0.003**
	Male	1.09 (0.58–2.01)	0.797
	HT	1 (0.51–1.98)	0.994
	DM	2.1 (1.17–3.76)	**0.013**
	eGFR <60	0.91 (0.44–1.90)	0.801
	EF <0.5	1.7 (0.91–3.19)	0.100
	Wellens' syndrome	0.47 (0.21–1.06)	0.070
	LM disease	0.81 (0.39–1.67)	0.560

Bold values indicate that the difference is statistically significant (*P* < 0.05).

## Data Availability

Access to the data is restricted because of third-party rights and patient privacy.
