# Correction to “Characteristics of Wellens’ Syndrome in the Current PCI Era: A Single‐Center Retrospective Study”

**DOI:** 10.1155/emmi/9838150

**Published:** 2026-08-19

L. Zhou, X. Gong, H. Chen, T. Dong, H. Cui, and H. Li, “Characteristics of Wellens’ Syndrome in the Current PCI Era: A Single‐Center Retrospective Study,” *Emergency Medicine International* 2023, no. 1 (2023): 8865553, https://doi.org/10.1155/2023/8865553.

In the above article, the authors did not explicitly state that the study was derived from a previously published patient cohort [1]. While the prior article was cited, this correction seeks to address the relationship between the two publications.

The data used in this study were obtained from the same institutional database (Cardiovascular Center of Beijing Friendship Hospital, 2017–2019) as the authors’ earlier publication [1]. The earlier study investigated Wellens’ syndrome in all NSTE‐ACS patients with culprit left anterior descending (LAD) vessels (*n* = 2,127), including both non‐ST‐segment elevation myocardial infarction (NSTEMI) and unstable angina. It focused broadly on incidence, risk factors, and long‐term prognosis across the entire NSTE‐ACS spectrum.

The present study is a secondary, prespecified subgroup analysis focusing exclusively on NSTEMI patients (*n* = 476), a higher‐risk subgroup with distinct biomarker profiles. This analysis provides novel and clinically relevant information that was not detailed or adequately powered in the original study, including

• Angiographic characteristics (e.g., higher proportion of single‐vessel disease)
• Early percutaneous coronary intervention (PCI) timing (≤ 24 hours from first medical contact)
• Cardiac readmission rates as a secondary outcome

The two studies address different research questions and patient populations. The overlap in study period, institution, and registry is fully acknowledged. The authors regret that this relationship was not clearly disclosed in the original manuscript.

The scientific conclusions of the original article remain unchanged.

We apologize for this error.
